# Retraction: *Aeromonas hydrophila* flagella glycosylation: Involvement of a lipid carrier

**DOI:** 10.1371/journal.pone.0324165

**Published:** 2025-05-09

**Authors:** The PLOS One Editors

After this article [1] was published, concerns were raised about Figs 2, 4, 7–9.

Specifically:

- In Fig 2A:
  - There are multiple areas of background patterns that appear more similar than would be expected when levels are adjusted.
  - Lanes St, 1 and 2 appear to be similar to lanes St, 1 and 2 in Fig 1B of [2].
- In Fig 4B:
  - Areas around the band in lane 2 appear to be discontinuous with the adjacent background when levels are adjusted.
  - Lanes 1 and 3 appear similar to lanes 2 and 3 in Fig 8B, despite being used to represent different experimental results.
- The Fig 7A panel 3 of this article [1] appears similar to the Fig 6 AH-3 panel in [3] and to the Fig 3F panel in [4] despite representing results from different strains or organisms.
- In Fig 8B:
  - There appears to be a vertical discontinuity between lanes 1 and 2.
  - When background levels are adjusted to improve contrast, there appear to be multiple areas with repetitive background noise patterns.
- In Fig 9A, panel 3 appears similar to the Fig 2E panel from [4], representing a different bacterium.

The corresponding author provided image data underlying the results presented in Figs 4B and 9A, stating that Fig 4B presents a composition of different lanes from the same gel. The corresponding author was unable to provide underlying image data for the results presented in Figs 2A, 7A, and 8B, but they confirmed that the panel presented in Fig 8B is incorrect. In the absence of original data, the concerns regarding these figures cannot be resolved.

In light of the above concerns, which question the reliability of the reported results, the *PLOS One* Editors retract this article.

SM, MW, and JMT did not respond to the final editorial decision. KMF, SMT, and RM either did not respond directly or could not be reached.

The Fig 7A panel 3 reports material from [3], published in 2011 by Microbiology Society, which is not offered under a CC-BY license and is therefore excluded from this article’s [1] license. The article [1] was republished on April 25, 2025, to note this exclusion in the Fig 7A legend and the article’s copyright statement.
